# Risk factors for comorbid hypertension and depression in the elderly: Evidence from the China health and retirement longitudinal study

**DOI:** 10.1097/MD.0000000000044004

**Published:** 2025-08-15

**Authors:** Shengshi Li, Ruihua Yang, Xuekui Liu, Xiangke Li

**Affiliations:** a Department of Cardiac Function, Xuzhou Central Hospital, Southeast University Affiliated Xuzhou Central Hospital, Xuzhou, Jiangsu Province, China; b Department of Central Laboratory, Xuzhou Central Hospital, Southeast University affiliated Xuzhou Central Hospital, Xuzhou, Jiangsu Province, China; c Department of Cardiac Function, Xuzhou Medical Science Research Institute, Xuzhou, Jiangsu Province, China.

**Keywords:** aged, depression, hypertension, risk factors

## Abstract

As the aging process accelerates, an increasing number of elderly individuals are experiencing psychological issues, particularly those with hypertension. Identifying the risk factors for comorbid hypertension and depression in the elderly is crucial for improving their quality of life. The China Health and Retirement Longitudinal Study is a survey conducted among older adults in China, collecting general data from middle-aged and elderly individuals, including age, gender, marital status, education level, smoking and drinking habits, and the presence of hypertension. The study also assesses participants’ mental health using the 10-item Center for Epidemiologic Studies-Depression Scale (CES-D10). A total of 11,519 individuals were included in this analysis. Among the 11,519 participants, 1671 were diagnosed with hypertension, while 9848 were not, resulting in a hypertension prevalence of 14.51%. A total of 4076 individuals scored ≥10 on the CES-D10, indicating a depression prevalence of 35.39%. Among those with hypertension, 704 were also diagnosed with depression, yielding a comorbidity rate of 42.13%, which is higher than the 34.13% observed in non-hypertensive participants. Further analysis identified age, smoking, widowhood or separation, and self-rated health status as independent risk factors for comorbid hypertension and depression. Hypertension have a higher risk of developing depression compared to the general population. Age, illiteracy, smoking, widowhood or separation are independent risk factors for comorbid hypertension and depression.

## 1. Introduction

Depression poses a significant and multifaceted threat to the physical and mental well-being, as well as the quality of life, of elderly individuals.^[1]^ According to findings from the World Mental Health Survey, approximately 5% of the global population has experienced at least 1 depressive episode during their lifetime.^[2]^ Globally, depression affects an estimated 350 million individuals, with reported lifetime prevalence rates of 28.6% for males and 29.8% for females.^[3]^ Data from the global burden of disease database highlight a concerning trend: between 1990 and 2019, the number of global depression cases increased by 59.28%. Projections indicate that by 2030, the total number of cases is expected to reach 108,868,019 for males and 164,886,024 for females.^[4]^ In China alone, there were 41 million reported cases of depression in 2019, reflecting a 31% increase since 1990.^[5]^ These figures underscore the escalating severity of depression on a global scale, with particularly alarming implications for rapidly aging populations such as those in China.

Depression serves as a crucial risk factor for hypertension and cardiovascular diseases, significantly augmenting the likelihood of coronary heart disease by a factor of 1.5 to 2.0.^[6]^ Individuals with depression exhibit a markedly higher risk of developing hypertension compared to those without depression. A meta-analysis has revealed that the overall prevalence of depression among hypertensive patients is 26.8% (95% confidence interval: 21.7%–32.3%).^[7]^ Hypertension is a type of psychosomatic disease that can induce anxiety and depressive emotional states.^[8]^ Conversely, anxiety and depression are significant contributing factors that may trigger hypertension.^[9]^ These 2 conditions interact with each other, leading to recurrent episodes and challenges in disease management. In patients already suffering from coronary heart disease, depression escalates the risk of myocardial infarction by 1.5 to 4.5 times.^[10]^ Many studies have reported on the prevalence of depression among hypertensive patients; however, the prevalence rates vary significantly across different countries and regions, particularly within middle-aged and elderly populations.

China, with its large population, has been experiencing a progressively accelerating aging process in recent years.^[11]^ Data indicates that by 2025, the number of individuals aged 60 and above in China will reach 300 million, and by 2027, the country will enter a phase of deep aging society.^[12]^ The substantial increase in the elderly population is poised to exert considerable pressure on the healthcare system. Consequently, focusing on the health of middle-aged and elderly individuals has become critically important.^[13]^ In this study, we utilized data from the China Health and Retirement Longitudinal Study (CHARLS) to investigate the epidemiology of comorbid hypertension and depression, as well as to identify associated risk factors among middle-aged and elderly populations in China.

## 2. Individuals and methods

### 2.1. Individuals

The data used in this study was derived from CHARLS, which adopted a random sampling strategy to collect nationally representative, high-quality microdata from Chinese households. This national baseline survey, conducted every 3 years since 2011, encompasses a total of 450 communities (including villages) across 150 counties spanning 28 provinces. Participants who met the following criteria were considered eligible for inclusion in this study: complete records of demographic information, lifestyle factors, and health behavior data; provision of information related to hypertension, responses to the CES-D10 depression questionnaire, and age over 45 years. Individuals who lacked any of the aforementioned data were excluded. After excluding 8297 participants, a total of 11,519 individuals were included in the analysis. Among these, 1671 participants were diagnosed with hypertension, of whom 704 also suffered from depression, while among the 9848 non-hypertensive participants, 3372 were found to have depression.

**Figure 1.**
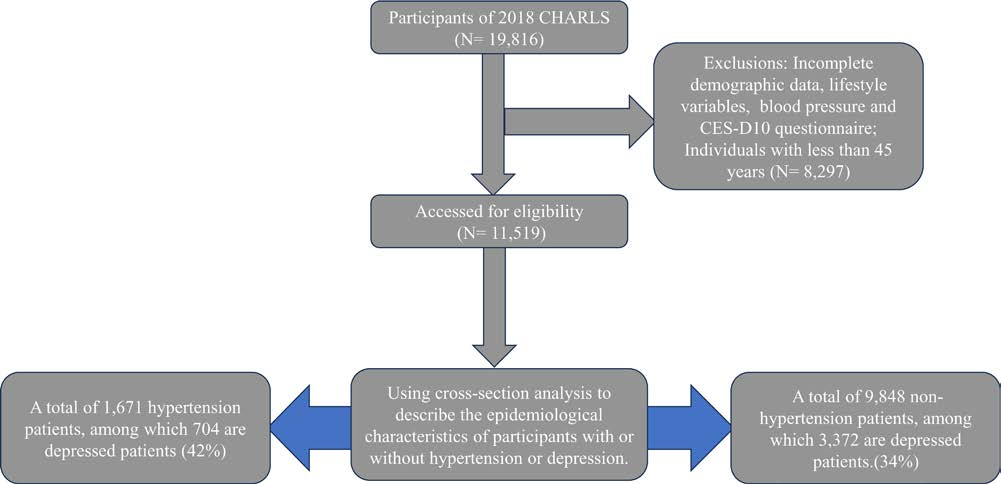
The flowchart of this study. CES-D10 = the10-item center for epidemiologic studies-depression scale, CHARLS = the China health and retirement longitudinal study.

The study was granted ethical approval by the Biomedical Ethics Committee at Peking University, and the fieldwork protocol was also approved (approval number: IRB00001052-11015). A detailed flowchart outlining the study design is provided in Figure 1.

### 2.2. Assessment of hypertension

In the CHARLS 2018 survey, participants were asked whether they had been diagnosed with hypertension by a doctor via the question: “Have you been diagnosed with Hypertension by a doctor?” Responses were recorded as either “Yes” or “No.” Additionally, participants were queried about their current treatments for hypertension or its complications with the question: “Are you now taking any of the following treatments to treat hypertension or its complications? Taking Chinese traditional medicine, taking Western modern medicine, other treatments?” Based on these responses, we determined whether participants currently had hypertension.

### 2.3. Assessment of depression

Depressive symptoms were assessed using the 10-item Center for Epidemiological Studies-Depression Scale (CES-D10), which is included in the CHARLS survey to evaluate whether participants exhibit symptoms of depression. The CES-D10 scale consists of 10 items: “I was bothered by things that usually don’t bother me”; “I had trouble keeping my mind on what I was doing”; “I felt depressed”; “I felt that everything I did was an effort”; “I felt fearful”; “My sleep was restless”; “I was happy”; “I felt lonely”; “I felt lonely”; and “I could not ‘get going.’” Each item is scored on a 4-point Likert scale with the following response options: “Rarely or none of the time (<1 day),” “Some or a little of the time (1–2 days),” “Occasionally or a moderate amount of time (3–4 days),” and “All of the time (5–7 days).”

In this scale, 2 items – “I felt hopeful about the future” and “I was happy” – are reverse-scored. The total score ranges from 0 to 30, with higher scores indicating more severe depressive symptoms. Participants were classified as having depressive symptoms if their CES-D10 score was 10 or higher. The Cronbach α coefficient of the CES-D10 scale in the Chinese population is 0.815, demonstrating its reliability as a self-rated measure of depressive symptoms.

### 2.4. Statistics methods

R4.4.1 statistical software was employed to manage and analyze the data. Participants were categorized into 2 groups based on the presence or absence of hypertension. χ^2^ tests was utilized to compare differences in gender, age, educational level, marital status, alcohol consumption, smoking habits, and self-rated health status between these 2 groups. Additionally, the prevalence of depression was analyzed across the groups.

To explore potential variations due to different age groups, participants were stratified into 8 age categories, each spanning 10 years. The prevalence rates of hypertension, depression, and comorbid hypertension and depression were then analyzed within various subgroups, including different age brackets, genders, marital statuses, and lifestyle habits. Univariate and multivariate logistic regression analyses were conducted to examine the associations between different risk factors and the co-occurrence of depression and hypertension. A *P*-value of <.05 was considered statistically significant.

## 3. Results

### 3.1. Clinical characteristics of participants in CHARLS 2018

In the CHARLS 2018 survey, a total of 19,816 individuals were investigated. After excluding participants younger than 45 years and those with incomplete data, the analysis included 11,519 elderly individuals from China. Among these participants, 1671 were diagnosed with hypertension, while 9848 were not.

Table 1 presents a comparison of demographic characteristics between the 2 groups, including age, gender, marital status, education level, self-rated health, smoking, and alcohol consumption. The findings indicate that compared to the non-hypertensive group, individuals with hypertension were older, had a lower proportion of females, and exhibited a higher smoking rate. Table S1, Supplemental Digital Content, https://links.lww.com/MD/P711 provides each item of the short depression scale, along with the distribution status of hypertensive and non-hypertensive populations for each item. The data indicates that except for the item “I felt hopeful about the future,” where there is no difference between the 2 groups, there are statistically significant differences between the 2 groups for the other 9 items.

**Table 1:** The characteristics of mid and old participants according to hypertension status.

	Hypertension		P
	Yes	No	
n	1671	9848	–
Age (mean, SD)	61.65 (9.35)	59.35 (9.01)	<.001
Gender (%)			
Female	779 (46.6)	5062 (51.4)	<.001
Male	892 (53.4)	4786 (48.6)	
Education (%)			
Illiterate	302 (18.1)	1663 (16.9)	.427
Did not finish primary school	347 (20.8)	2023 (20.5)	
Elementary school	396 (23.7)	2296 (23.3)	
Middle school	398 (23.8)	2475 (25.1)	
High school	141 (8.4)	926 (9.4)	
Vocational school	54 (3.2)	234 (2.4)	
Associate degree	19 (1.1)	142 (1.4)	
Above bachelor’s degree	14 (0.8)	89 (0.9)	
Marital status (%)			
Married and live with spouse	1317 (78.8)	8072 (82.0)	.015
Separated	130 (7.8)	755 (7.6)	
Divorced	27 (1.6)	126 (1.3)	
Widowed	188 (11.3)	850 (8.6)	
Never married	9 (0.5)	45 (0.5)	
Self-report health status (%)			
Very good	143 (8.6)	1560 (15.8)	<.001
Good	182 (10.9)	1516 (15.4)	
Fair	824 (49.3)	4906 (49.8)	
Poor	409 (24.5)	1451 (14.7)	
Very poor	113 (6.8)	414 (4.2)	
Smoking status (%)			
No	867 (51.8)	5644 (57.3)	<.001
Yes	152 (9.1)	414 (4.2)	
Refused	652 (39.1)	3790 (38.5)	
Drinking status (%)			
Drink more than once a month	478 (28.6)	2844 (28.9)	.488
Drink but less than once a month	128 (7.7)	834 (8.5)	
None of these	1065 (63.7)	6167 (62.6)	

SD = standard deviation.

### 3.2. Epidemiological features of depression, hypertension, and comorbid depression and hypertension among middle-aged and elderly individuals in China

The findings reveal that with increasing age, there is a corresponding rise in the prevalence of hypertension, depression, and their comorbidity. Specifically, in individuals aged over 70 years, the prevalence of hypertension surpasses 15%, the prevalence of depression reaches more than 35%, and the comorbidity rate of depression and hypertension stands at 10%.

**Figure 2.**
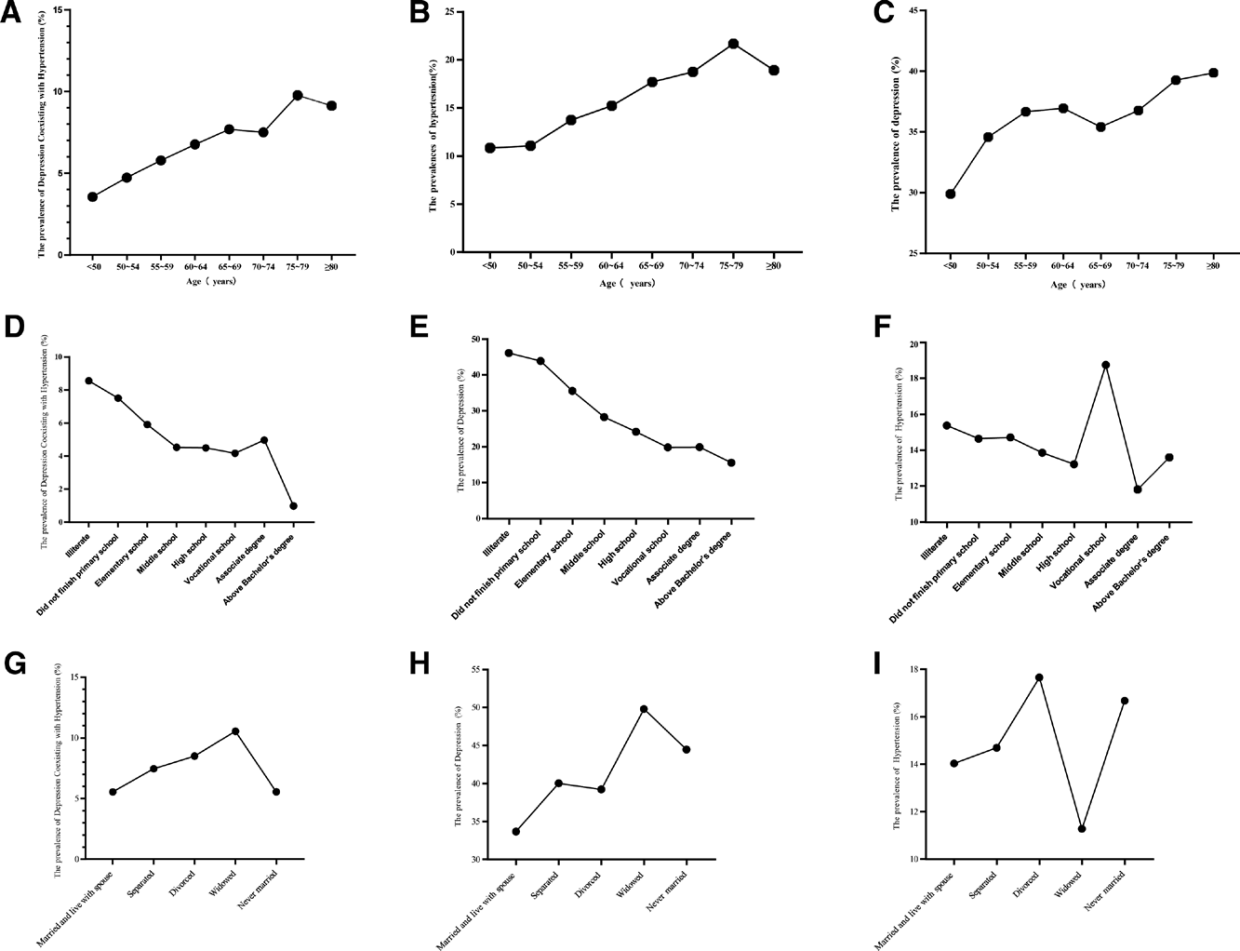
Age, educational levels and marital status for hypertension, depression, and comorbid hypertension and depression. (A) the prevalence of comorbid hypertension and depression in different age levels; (B) The prevalence of hypertension in different age levels; (C) the prevalence of depression in different age levels; (D) the prevalence of comorbid hypertension and depression in different education levels; (E) the prevalence of hypertension in different education levels; (F) the prevalence of depression in different education levels (G) the prevalence of comorbid hypertension and depression in different marital status; (H) The prevalence of hypertension in different marital status; (I) the prevalence of depression in different marital status.

In addition to age, educational levels and marital status as significant risk factors for these conditions. The analysis demonstrates an inverse relationship between educational level and the prevalence of hypertension, depression, and their comorbidity, with higher education levels associated with lower prevalence rates. Among different marital statuses, widowed individuals exhibit the highest prevalence of comorbid depression and hypertension, whereas divorced individuals display the highest prevalence of hypertension (Fig. 2).

**Figure 3.**
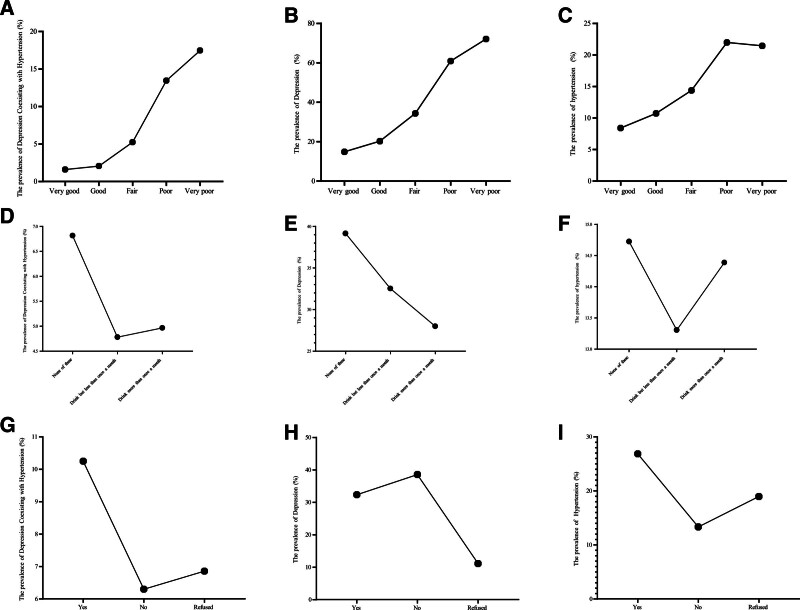
Smoking status, drinking status and self-report health status for hypertension, depression and comorbid hypertension and depression. (A) The prevalence of comorbid hypertension and depression in different self-report health status; (B) the prevalence of hypertension in different self-report health status; (C) the prevalence of depression in different self-report health status; (D) the prevalence of comorbid hypertension and depression in different drinking status; (E) the prevalence of hypertension in different drinking status; (F) the prevalence of depression in different drinking status; (G) the prevalence of comorbid hypertension and depression in different smoking status; (H) the prevalence of hypertension in different smoking status; (I) the prevalence of depression in different smoking status.

Lifestyle factors such as smoking and alcohol consumption were also identified as risk factors for depression and hypertension. As demonstrated in Figure 3, nonsmoking individuals exhibited the lowest prevalence of comorbid depression and hypertension, whereas non-drinking individuals showed the highest prevalence of these comorbid conditions.

Self-reported health status refers to the state in which respondents assess whether they are healthy or not. In this survey, respondents can choose from 5 states: “Very Good,” “Good,” “Fair,” “Poor,” and “Very Poor” to describe their health status. The data indicates that among the respondents, the prevalence rate of depression accompanied by hypertension is the lowest in the group reporting their health status as “Very Good.” In contrast, in the group considering their health as “Very Poor,” the prevalence rate of depression combined with hypertension is as high as 17.5%.

### 3.3. Risk factors for depression comorbid with hypertension in the Chinese population

Univariate and multivariate logistic regression analyses were performed to identify independent risk factors for the comorbidity of depression and hypertension. Table 2 shows that in the univariate binomial logistic regression, factors such as female gender, age over 55 years, low educational level (those who have not completed primary school or are illiterate), smoking, separation or widowhood, and poor self-perceived health status are risk factors for the co-occurrence of depression and hypertension. Further multivariate binomial logistic regression analysis reveals that age over 55 years, illiteracy, smoking, separation or widowhood, and poor self-perceived physical condition are independent risk factors for the co-occurrence of depression and hypertension.

**Table 2:** Logistic regression to explore the risk factor of comorbid hypertension and depression.

Variables	Univariate		Multivariate	
	OR (95% CI)	*P*	OR (95% CI)	*P*
Gender				
Female	1.237 (1.062–1.443)	.006	1.064 (0.826–1.375)	.632
Age (year)				
<50	1	–	1	–
50–55	1.349 (0.976–1.888)	.075	1.266 (0.912–1.780)	.166
55–60	1.666 (1.202–2.338)	.003	1.553 (1.112–2.195)	.011
60–65	1.970 (1.434–2.746)	<.001	1.623 (1.170–2.283)	.004
65–70	2.263 (1.640–3.163)	<.001	1.704 (1.222–2.408)	.002
70–75	2.204 (1.530–3.192)	<.001	1.617 (1.109–2.372)	.013
75–80	2.940 (1.967–4.390)	<.001	2.022 (1.325–3.081)	.001
≥80	2.727 (1.666–4.375)	<.001	1.929 (1.143–3.190)	.012
Education				
Illiterate	1	–	1	–
Did not finish primary school	0.868 (0.697–1.083)	.209	1.015 (0.805–1.280)	.898
Elementary school	0.671 (0.536–0.841)	.005	0.848 (0.665–1.081)	.183
Middle school	0.506 (0.399–0.642)	<.001	0.757 (0.583–0.982)	.036
High school	0.504 (0.358–0.694)	<.001	0.798 (0.555–1.132)	.216
Vocational school	0.465 (0.242–0.812)	.012	0.752 (0.386–1.339)	.365
Associate degree	0.559 (0.248–1.087)	.117	1.121 (0.488–2.241)	.766
Above Bachelor’s degree	0.105 (0.006–0.474)	.024	0.228 (0.013–1.051)	.144
Smoking status				
Yes	1	–	–	–
No	0.588 (0.444–0.793)	<.001	0.517 (0.365–0.739)	.002
Refused	0.491 (0.366–0.669)	<.001	0.465 (0.341–0.643)	<.001
Marital status (%)				
Married and live with spouse	1	–	–	–
Separated	1.372 (1.042–1.777)	.019	1.464 (1.103–1.916)	.006
Divorced	1.580 (0.846–2.701)	.118	1.533 (0.802–2.698)	.164
Widowed	1.835 (1.460–2.284)	<.001	1.317 (1.024–1.679)	.029
Never married	1.001 (0.243–2.732)	.998	0.840 (0.201–2.698)	.775
Self-report health status (%)				
Very good	1	–	–	–
Good	1.306 (0.789–2.185)	.301	1.294 (0.781–2.169)	.319
Fair	3.429 (2.350–5.222)	<.001	3.306 (2.263–5.039)	<.001
Poor	9.638 (6.566–14.745)	<.001	8.464 (5.745–12.986)	<.001
Very poor	13.128 (8.561–20.784)	<.001	11.445 (7.431–18.191)	<.001
Drinking status (%)				
Drink more than once a month	1	–	1	–
Drink but less than once a month	0.961 (0.680–1.331)	.815	0.919 (0.644–1.288)	.633
None of these	1.399 (1.170–1.682)	<.001	1.064 (0.867–1.312)	.553

CI = confidence interval, OR = odds ratio.

## 4. Discussion

In this large-scale study involving 11,519 middle-aged and elderly individuals in China, we identified a depression prevalence of 35.4% across all participants and 42.1% among those with hypertension. Independent risk factors for comorbid hypertension and depression included age over 55 years, illiteracy, smoking, being separated or widowed, and poor self-perceived physical health.

Depression has emerged as one of the most critical public health challenges among middle-aged and elderly populations globally.^[14]^ Epidemiological studies reveal that the prevalence of major depressive disorder in individuals aged 65 and older ranges from 1% to 4% in community-dwelling adults.^[15]^ However, this figure escalates dramatically to 25% among older adults with chronic medical conditions, with women exhibiting higher prevalence compared to men. The condition is strongly associated with diminished quality of life, manifesting in elevated rates of bedridden cases and suicide mortality. For instance, in the United States, the suicide rate among depressed older adults is twice as high as that of the general population.^[16]^ Furthermore, depression contributes to increase in all-cause mortality, likely mediated through its bidirectional relationships with comorbidities such as hypertension, diabetes, stroke, and cancer.^[17]^

Hypertension is a common comorbidity in patients with depression. Research has shown that depression is one of the factors contributing to increased blood pressure and vascular endothelial damage.^[18]^ Zhang Haijuan et al conducted 24-hour blood pressure monitoring and vascular endothelial function measurements in patients with depression. They found that indices of blood pressure variability (including 24-hour mean systolic blood pressure, 24-hour mean diastolic blood pressure, 24-hour systolic blood pressure coefficient of variation, and 24-hour diastolic blood pressure coefficient of variation) were significantly higher in the depression group compared to the control group.^[19]^ Additionally, the vascular endothelial function index was lower in the depression group, suggesting an increase in vascular endothelial damage.

Age and illiteracy have been confirmed by most studies as significant risk factors for hypertension comorbid with depression. As individuals age, their risk of developing hypertension increases. Concurrently, heightened concerns about the well-being and quality of life of family members (such as children and other immediate relatives) associated with aging may exacerbate depressive symptoms.^[20]^ Among illiterate individuals, lower educational attainment hinders their ability to develop personal skills, and they may lack the knowledge or means to access mental health resources and support. Additionally, lower education levels may contribute to poorer quality of life and weaker social support systems, further elevating the risk of depression.^[21]^

The study by Matthew et al showed that, after adjusting for other factors, being widowed is an independent risk factor for both hypertension and depression (hazard ratio 1.38, 95% CI: 1.11–1.71),^[22]^ which aligns with our findings. We speculate that the psychological stress and emotional distress resulting from widowhood can impact mental health, which in turn affects physical health. Widowhood also leads to lifestyle changes, such as alterations in dietary habits and exercise routines, which may increase the risk of developing hypertension and depression among affected individuals.

Hypertension and depression share certain comorbid foundations, as both conditions are associated with dysregulation of the hypothalamic-pituitary-adrenal (HPA) axis. Chengwen Wang et al found^[23]^ that HPA axis dysfunction increases cortisol secretion and circulating pro-inflammatory cytokines, which collectively impair blood pressure regulation and contribute to depression onset. In comorbid cases, HPA axis dysregulation appears more pronounced than in patients with either condition alone.

Low-grade inflammation serves as a critical common factor in the comorbidity of hypertension and depression. Jing Wang et al identified^[24]^ elevated inflammatory markers in hypertensive patients with concurrent anxiety and depression, indicating that chronic inflammation exacerbates both conditions. Xudong Meng et al underscore^[25]^ that inflammatory markers such as C-reactive protein (CRP) and interleukin-6 (IL-6) are elevated in patients with both hypertension and depression, indicating chronic inflammation as a key mediator. A cross-sectional study by Jiannan Feng demonstrated that the neutrophil-to-lymphocyte ratio, a systemic inflammation index, significantly predicted moderate-to-severe depressive symptoms (OR: 1.441, 95% CI: 1.017–2.042, *P* = .04) in patients undergoing maintenance hemodialysis.^[26]^ Similarly, Marco La Verde identified^[27]^ chronic low-grade inflammation-marked by elevated CRP and IL-6 levels-as a critical factor in the development and worsening of depressive symptoms. These findings collectively highlight low-grade inflammation’s role in mediating comorbidity through multifactorial pathways, necessitating integrated therapeutic strategies that target both physiological inflammation and psychological symptoms for effective management.

This study has several limitations. First, the data analyzed were derived from the CHARLS, a large-scale survey. Although the sample size was substantial, the cross-sectional design precludes establishing causal relationships between identified factors (e.g., age, smoking, widowhood, or being separated or widowed) and the comorbid hypertension and depression. Second, the research focused on middle-aged and older adults, whose unique life stages may involve depression-related mechanisms that were not accounted for in the current analysis.

In summary, this study found that in middle-aged and elderly populations in China, individuals with hypertension have a higher risk of developing depression compared to the general population. Age, illiteracy, smoking, widowhood or separation are independent risk factors for comorbid hypertension and depression. Healthcare providers should pay attention to the living conditions of middle-aged and elderly individuals.

## Acknowledgments

The authors would like to thank all participants for their cooperation and sample contributions and all the reviewers who participated in the review, as well as MJEditor (www.mjeditor.com) for providing English editing services during the preparation of this manuscript.

## Author contributions

**Conceptualization:** Xiangke Li.

**Data curation:** Ruihua Yang, Xuekui Liu.

**Formal analysis:** Ruihua Yang, Xuekui Liu.

**Visualization:** Shengshi Li.

**Writing – original draft:** Shengshi Li.

**Writing – review & editing:** Xiangke Li.

## Supplementary Material



## References

[1] McCarron RM Shapiro B Rawles J Luo J. Depression. Ann Intern Med. 2021;174:ITC65–80. 33971098 10.7326/AITC202105180

[2] Alonso J Liu Z Evans-Lacko S; WHO World Mental Health Survey Collaborators. Treatment gap for anxiety disorders is global: results of the world mental health surveys in 21 countries. Depress Anxiety. 2018;35:195–208. 29356216 10.1002/da.22711 PMC6008788

[3] McGrath JJ Al-Hamzawi A Alonso J; WHO World Mental Health Survey Collaborators. Age of onset and cumulative risk of mental disorders: a cross-national analysis of population surveys from 29 countries. Lancet Psychiatry. 2023;10:668–81. 37531964 10.1016/S2215-0366(23)00193-1 PMC10529120

[4] Zhang Y Chen Y Ma L. Depression and cardiovascular disease in elderly: current understanding. J Clin Neurosci. 2018;47:1–5. 29066229 10.1016/j.jocn.2017.09.022

[5] He J Chang L Zhang L Wu W Zhuo D. Effect of probiotic supplementation on cognition and depressive symptoms in patients with depression: a systematic review and meta-analysis. Medicine (Baltimore). 2023;102:e36005. 38013351 10.1097/MD.0000000000036005 PMC10681621

[6] Bergantin LB. Depression rises the risk of hypertension incidence: discussing the link through the Ca2+/cAMP signalling. Curr Hypertens Rev. 2020;16:73–8. 30648516 10.2174/1573402115666190116095223

[7] Gan Q Yu R Lian Z Yuan Y Li Y Zheng L. Unraveling the link between hypertension and depression in older adults: a meta-analysis. Front Public Health. 2023;11:1302341. 38074728 10.3389/fpubh.2023.1302341 PMC10704466

[8] Zhang H Zhang X Jiang X. Mindfulness-based intervention for hypertension patients with depression and/or anxiety in the community: a randomized controlled trial. Trials. 2024;25:299. 38698436 10.1186/s13063-024-08139-0 PMC11529483

[9] Herrmann-Lingen C alʼAbsi M. Exploring the association of hypertension with risk for depression: evidence for tamed neurobehavioral arousal versus central emotional dysregulation. Psychosom Med. 2018;80:504–7. 29851869 10.1097/PSY.0000000000000611

[10] Sayar K Gulec H Gokce M Ak I. Heart rate variability in depressed patients. Bull Clin Psychopharmacol. 2002;12:130–3.

[11] Cai Y Song W Li J. The landscape of aging. Sci China Life Sci. 2022;65:2354–454. 36066811 10.1007/s11427-022-2161-3 PMC9446657

[12] Ren Z. Report on aging in China. Develop Res. 2023;40:9.

[13] Li C Chen Z Khan MM. Bypassing primary care facilities: health-seeking behavior of middle age and older adults in China. BMC Health Serv Res. 2021;21:895. 34461884 10.1186/s12913-021-06908-0 PMC8406824

[14] Vos T Flaxman AD Naghavi M. Years lived with disability (YLDs) for 1160 sequelae of 289 diseases and injuries 1990-2010: a systematic analysis for the Global Burden of Disease Study 2010. Lancet. 2012;380:2163–96. 23245607 10.1016/S0140-6736(12)61729-2 PMC6350784

[15] Stubbs B Stubbs J Gnanaraj SD Soundy A. Falls in older adults with major depressive disorder (MDD): a systematic review and exploratory meta-analysis of prospective studies. Int Psychogeriatr. 2016;28:23–9. 26234532 10.1017/S104161021500126X

[16] Katz IR Rogers MP Lew R; Li+ plus Investigators. Lithium treatment in the prevention of repeat suicide-related outcomes in veterans with major depression or bipolar disorder: a randomized clinical trial. JAMA Psychiatry. 2022;79:24–32. 34787653 10.1001/jamapsychiatry.2021.3170 PMC8600458

[17] Chu WM Liao WC Li CR. Late-career unemployment and all-cause mortality, functional disability and depression among the older adults in Taiwan: a 12-year population-based cohort study. Arch Gerontol Geriatr. 2016;65:192–8. 27070503 10.1016/j.archger.2016.03.020

[18] Plante GE. Depression and cardiovascular disease: a reciprocal relationship. Metabolism. 2005;54(5 Suppl 1):45–8. 15877313 10.1016/j.metabol.2005.01.013

[19] Zhang H Tian X Zhai Y Yao L Wu Y. Effects of anxiety and depression on blood pressure variability and vascular endothelial cell function in patients with hypertension. China Med. 2023;18:1140–4.

[20] Baird B Oh KM Douglas C Weinstein AA. Health literacy, depression literacy, and depression among older Korean Americans. J Health Commun. 2019;24:525–35. 31244411 10.1080/10810730.2019.1632395

[21] Albasara SA Haneef MS Zafar M Moinuddin KG. Depression and associated risk factors among hypertensive patients in primary health care centers in Dammam, Kingdom of Saudi Arabia. Pan Afr Med J. 2021;38:278. 34122705 10.11604/pamj.2021.38.278.27133 PMC8179995

[22] Pantell MS Prather AA Downing JM Gordon NP Adler NE. Association of social and behavioral risk factors with earlier onset of adult hypertension and diabetes. JAMA Netw Open. 2019;2:e193933. 31099868 10.1001/jamanetworkopen.2019.3933 PMC6537925

[23] Wang C Li S Song Y Yuan X Zhu H Yu B. Prospective association of comorbid hypertension and depressive symptoms with C-reactive protein in older adults. J Affect Disord. 2024;354:286–92. 38484887 10.1016/j.jad.2024.03.066

[24] Wang J Chen J. Changes in serum inflammatory factors and monoamine neurotransmitter levels in patients with hypertension complicated with anxiety and depression. Chin J Hyperten. 2023;31:4.

[25] Meng X Han L Fu J Hu C Lu Y. Associations between metabolic syndrome and depression, and the mediating role of inflammation: based on the NHANES database. J Affect Disord. 2025;375:214–21. 39862983 10.1016/j.jad.2025.01.108

[26] Feng J Lu X Li H Wang S. High neutrophil-to-lymphocyte ratio is a significant predictor of depressive symptoms in maintenance hemodialysis patients: a cross-sectional study. BMC Psychiatry. 2022;22:313. 35505395 10.1186/s12888-022-03963-7 PMC9063198

[27] La Verde M Luciano M Fordellone M. Postpartum depression and inflammatory biomarkers of neutrophil-lymphocyte ratio, platelet-lymphocyte ratio, and monocyte-lymphocyte ratio: a prospective observational study. Gynecol Obstet Invest. 2024;89:140–9. 38346412 10.1159/000536559

