# Supplementary material for: Risk factors for comorbid hypertension and depression in the elderly: Evidence from the China health and retirement longitudinal study
Source: Medicine (Baltimore). 2025 Aug 15;104(33):e44004. doi: 10.1097/MD.0000000000044004 (PMC12367006; doi:10.1097/MD.0000000000044004)
Supplement: Supplementary file 1 [file medi-104-e44004-s001.docx]

Supplement Table 1

|  | Hypertesion | |  |
| --- | --- | --- | --- |
|  | Yes | No | P |
| n | 1,671 | 9,848 |  |
| **I was bothered by things that usually don't bother me.** |  |  | 0.008 |
| Rarely or none of the time (less than 1 day) | 808 (48.4) | 4987 ( 50.6) |  |
| Some or a little of the time (1‐2 days) | 365 (21.8) | 2178 ( 22.1) |  |
| Occasionally or a moderate amount of time (3‐4 days) | 238 (14.2) | 1448 ( 14.7) |  |
| All of the time (5‐7 days) | 260 (15.6) | 1235 ( 12.5) |  |
| **I had trouble keeping my mind on what I was doing** |  |  | <0.001 |
| Rarely or none of the time (less than 1 day) | 798 (47.8) | 5094 ( 51.7) |  |
| Some or a little of the time (1‐2 days) | 347 (20.8) | 2099 ( 21.3) |  |
| Occasionally or a moderate amount of time (3‐4 days) | 272 (16.3) | 1502 ( 15.3) |  |
| All of the time (5‐7 days) | 254 (15.2) | 1153 ( 11.7) |  |
| **I felt depressed.** |  |  | <0.001 |
| Rarely or none of the time (less than 1 day) | 794 (47.5) | 5103 ( 51.8) |  |
| Some or a little of the time (1‐2 days) | 354 (21.2) | 2122 ( 21.5) |  |
| Occasionally or a moderate amount of time (3‐4 days) | 273 (16.3) | 1511 ( 15.3) |  |
| All of the time (5‐7 days) | 250 (15.0) | 1112 ( 11.3) |  |
| **I felt that everything I did was an effort.** |  |  | <0.001 |
| Rarely or none of the time (less than 1 day) | 815 (48.8) | 5368 ( 54.5) |  |
| Some or a little of the time (1‐2 days) | 279 (16.7) | 1784 ( 18.1) |  |
| Occasionally or a moderate amount of time (3‐4 days) | 269 (16.1) | 1313 ( 13.3) |  |
| All of the time (5‐7 days) | 308 (18.4) | 1383 ( 14.0) |  |
| I felt hopeful about the future. |  |  | 0.329 |
| Rarely or none of the time (less than 1 day) | 693 (41.5) | 4219 ( 42.8) |  |
| Some or a little of the time (1‐2 days) | 270 (16.2) | 1672 ( 17.0) |  |
| Occasionally or a moderate amount of time (3‐4 days) | 227 (13.6) | 1324 ( 13.4) |  |
| All of the time (5‐7 days) | 481 (28.8) | 2633 ( 26.7) |  |
| **I felt fearful.** |  |  | 0.001 |
| Rarely or none of the time (less than 1 day) | 1266 (75.8) | 7840 ( 79.6) |  |
| Some or a little of the time (1‐2 days) | 175 (10.5) | 968 ( 9.8) |  |
| Occasionally or a moderate amount of time (3‐4 days) | 122 ( 7.3) | 588 ( 6.0) |  |
| All of the time (5‐7 days) | 108 ( 6.5) | 452 ( 4.6) |  |
| **My sleep was restless.** |  |  | <0.001 |
| Rarely or none of the time (less than 1 day) | 722 (43.2) | 4809 ( 48.8) |  |
| Some or a little of the time (1‐2 days) | 280 (16.8) | 1648 ( 16.7) |  |
| Occasionally or a moderate amount of time (3‐4 days) | 269 (16.1) | 1443 ( 14.7) |  |
| All of the time (5‐7 days) | 400 (23.9) | 1948 ( 19.8) |  |
| **I was happy.** |  |  | 0.028 |
| Rarely or none of the time (less than 1 day) | 781 (46.7) | 4913 ( 49.9) |  |
| Some or a little of the time (1‐2 days) | 315 (18.9) | 1904 ( 19.3) |  |
| Occasionally or a moderate amount of time (3‐4 days) | 242 (14.5) | 1274 ( 12.9) |  |
| All of the time (5‐7 days) | 333 (19.9) | 1757 ( 17.8) |  |
| **I felt lonely.** |  |  | <0.001 |
| Rarely or none of the time (less than 1 day) | 1086 (65.0) | 7046 ( 71.5) |  |
| Some or a little of the time (1‐2 days) | 219 (13.1) | 1194 ( 12.1) |  |
| Occasionally or a moderate amount of time (3‐4 days) | 171 (10.2) | 812 ( 8.2) |  |
| All of the time (5‐7 days) | 195 (11.7) | 796 ( 8.1) |  |
| **I could not "get going."** |  |  | <0.001 |
| Rarely or none of the time (less than 1 day) | 1261 (75.5) | 7906 ( 80.3) |  |
| Some or a little of the time (1‐2 days) | 156 ( 9.3) | 817 ( 8.3) |  |
| Occasionally or a moderate amount of time (3‐4 days) | 132 ( 7.9) | 617 ( 6.3) |  |
| All of the time (5‐7 days) | 122 ( 7.3) | 508 ( 5.2) |  |
| Total-scores | 8.00 [3.00, 14.00] | 7.00 [3.00, 12.00] | <0.001 |
| Total-scores >= 10 |  |  | <0.001 |
| Yes | 704(42.1) | 3372(34.2) |  |
| No | 967(57.9) | 6476(65.8) |  |
